# Depression and Impulsivity Self-Assessment Tools to Identify Dopamine Agonist Side Effects in Patients With Pituitary Adenomas

**DOI:** 10.3389/fendo.2020.579606

**Published:** 2020-10-27

**Authors:** José Miguel Hinojosa-Amaya, Nathaniel Johnson, Christina González-Torres, Elena V. Varlamov, Christine G. Yedinak, Shirley McCartney, Maria Fleseriu

**Affiliations:** ^1^Department of Medicine (Endocrinology Diabetes and Clinical Nutrition) and Neurological Surgery, and Northwest Pituitary Center, Oregon Health & Science University, Portland, OR, United States; ^2^Pituitary Clinic, Endocrinology Division, Department of Medicine, Hospital Universitario “Dr. José E. González, ” Universidad Autónoma de Nuevo León, Monterrey, Mexico; ^3^Department of Psychiatry, Tecnologico de Monterrey, Escuela de Medicina y Ciencias de la Salud, Monterrey, Mexico

**Keywords:** dopamine agonist, depression, impulse control (pathology) disorders, impulsivity, prolactinoma, pituitary tumor, patient health questionnaire, hyperprocalctinemia

## Abstract

**Background:** Dopamine agonists (DA) are the first line therapy for prolactinoma and symptomatic hyperprolactinemia; use as an adjuvant treatment for acromegaly and Cushing's disease is rare. Some patients develop *de novo* psychiatric symptoms or have exacerbation of pre-existing conditions during DA therapy. A practical, clinically sensitive depression and impulse control disorders (ICD; particularly hypersexuality and gambling disorders) detection tool is important for identifying at risk patients. The Barratt Impulsivity Scale (BIS-11) and the 9-item Patient Health Questionnaire (PHQ-9) are sensitive in identifying impulsivity and depression.

**Objective:** Detail use of the BIS-11 and PHQ-9 as screening tools for depression and ICD in patients with pituitary disease at a high-volume academic pituitary center.

**Methods:** DA-treated and naïve patients with pituitary disease were included. Patients with a known history of depression or psychiatric disorder were excluded. PHQ-9 standardized interpretation criteria were utilized to classify depression severity. For BIS-11, threshold was established based on previous studies. Statistical analysis was with SPSS version 25.

**Results:** Seventy-six DA-treated and 27 naïve patients were included. Moderate and moderately severe depression were more prevalent in DA-treated patients; severe depression only found in DA-treated patients. A normal BIS-11 score was noted in 76.69%; higher scores (not significant) were noted in DA-treated patients. There was a positive correlation between higher BIS-11 and PHQ-9 scores; higher in DA-treated patients (*r* = 0.52, *p* < 0.001) than DA-naïve patients. Patients with BIS-11 scores ≥60 were younger and received lower cumulative DA doses compared to patients with BIS scores <60. There was no association between male sex and BIS-11 ≥60 and male sex did not increase the odds of increased scores (OR = 0.66, CI95% 0.25–1.76, *p* = 0.41). No significant difference was found for macroadenoma, prolactin levels, testosterone levels, hypogonadism, testosterone replacement in men, and increased impulsivity or depression scores.

**Conclusion:** Use of PHQ-9 and BIS-11 is practical for routine screening of depression and ICD during outpatient pituitary clinic visits for patients with pituitary disease both naïve to treatment and during DA therapy. We recommend close follow-up after initiation of DA therapy for younger patients, regardless of dose.

## Introduction

Prolactinomas (lactotroph adenomas) are the most frequently diagnosed functioning pituitary adenoma (1, 2). In healthy individuals, prolactin synthesis and secretion are inhibited by a tonic secretion of dopamine through the portal system, which activates dopamine type 2 (D2) receptors on the lactotroph cells (3, 4). Most prolactinomas retain dopamine-induced inhibition of hormone secretion, although some may be resistant (1, 5).

Dopamine agonists (DA) are the most effective treatment for prolactinomas and are a first-line therapy, causing both inhibition of prolactin secretion and pituitary tumor shrinkage (1, 6). Cabergoline and bromocriptine are the most widely used agents and are approved by both the US Food and Drug Administration (FDA) and the European Medicines Agency (EMA), while quinagolide is approved in just some countries (7). These medications are also used to treat symptomatic non-tumoral hyperprolactinemia, and more rarely as an adjuvant treatment for acromegaly and Cushing's disease (8, 9).

Concerns about the psychiatric effects of DA surfaced after isolated cases of psychosis, depression, hypersexuality, impulse control disorders (ICD) and other psychiatric symptoms were reported (7). Psychiatric effects of DA are theorized to be caused by multiple complex mechanisms, which include cross-stimulation of D3 receptors expressed on the meso-corticolimbic dopaminergic pathway, central nervous system dopamine depletion in hyperprolactinemia followed by sudden replacement and recovery of normal levels of gonadal sex steroids, especially testosterone, after central hypogonadism (7, 10). It is controversial whether hyperprolactinemia *per se* has a role (7). Of major concern is the co-existence of ICD with depression, which may cause a greater risk of suicidality (11).

There is a lack of uniform tools that can be used to identify depression and ICD prevalence in patients with DA-treated prolactinomas; most requiring a long and formal counseling appointment (as discussed below) and are not suitable for routine evaluation during pituitary clinic office visits. The Barratt Impulsivity Scale (BIS-11) is a validated self-reporting scale that is widely used and can easily be applied to identify ICD. BIS-11 has been used previously in patients with prolactinomas (12). The Patient Health Questionnaire 9 (PHQ-9) is a validated, 10-item self-reporting survey that identifies and classifies depression severity (13) including suicidal thoughts (14) with high sensitivity. Both questionnaires can be reviewed and interpreted in minutes, making them practical tools that may be used for routine screening of ICD and depression in DA-treated patients with pituitary diseases during office visits. Early detection of these disorders and discontinuation of DA therapy can mitigate potentially harmful consequences (15).

Here we describe, for the first time, use of both BIS-11 and PHQ-9 as screening tools for depression and ICD in DA-treated patients at a high-volume Pituitary Center.

## Methods

### Study Design and Patients

Consecutive patients from a pituitary registry who presented with a pituitary tumor and were treated with a DA in a Pituitary Clinic (years 2018 to 2019) were retrospectively reviewed for this cross-sectional study. Naïve (non-functioning or functioning pituitary adenoma; final diagnosis established after the survey date) to treatment patients were also reviewed. All patients had privately completed both BIS-11 and PHQ-9 as part of a standard clinic visit. Scores were calculated and interpreted during the same visit, and any abnormal results warranted further evaluation, DA medication adjustment, and, if necessary, an immediate referral to psychiatry. Patients who did not complete BIS-11 and PHQ-9 or had incomplete responses were excluded from analysis. Patients with a previous known diagnosis of depression, ICD, other psychiatric diseases or if on any anti-depressant, anti-psychotic, or psychotropic medication were also not given the surveys and excluded from this analysis. The pituitary registry is IRB-approved with a waiver of informed consent.

Patients (DA-treated and treatment naïve) were compared with regards to age, sex, presence of macroadenoma, type of pituitary tumor, prolactin level, testosterone level, hypogonadism, and testosterone replacement in men at the time of evaluation. A detailed analysis was undertaken comparing results of the PHQ-9 and BIS-11 between DA-treated and treatment naïve patients.

### Patient Health Questionnaire 9

PHQ-9 is a 9-item depression scale. Patients are asked to report specific depressive symptoms and frequency experienced in the previous 2 weeks on a 4-point scale from “not at all” to “nearly every day.” We used standardized interpretation criteria to classify individual patient's depression as minimal (<5 points), mild (5–9 points), moderate (10–14 points), moderately severe (15–19 points), and severe (≥20 points). The PHQ-9 criteria for major depression and other depressive disorders is shown in Supplementary Table 1. Special attention was paid to frequency indicated by item 9, which evaluates passive thought of death or self-injury within the previous 2 weeks and was used to screen for suicide risk (14). Results were categorized for major depression and other depressive disorders (by dichotomic presence or absence), as depression severity with ordinal variables and a numeric total score.

### Barratt Impulsivity Scale-11

The BIS-11 questionnaire is a 30-item screening tool for ICD. It includes items that assess impulsiveness with respect to factors such as; attention, motor, self-control, cognitive complexity, perseverance, cognitive instability, and non-planning impulsiveness. Patients are asked to score their average response to each item on a 4-point scale from “rarely/never” to “almost always/always,” thereby allowing for assessment of impulsiveness subscales; attention (AI), motor (MI), and non-planning (NPI), which also have several components, and a total score.

We assessed two different cut-offs for screening ICD based on the mean score of ICD patients evaluated in previous studies; 60 points based on prolactinoma patients diagnosed with ICD (16) and 65 points corresponding to ICD patients without pituitary disease but with gambling disorders (17).

### Dopamine Agonist Dosing Calculations

Patient cumulative cabergoline dose was estimated by multiplying the current cabergoline dose by the length of treatment in years, if the initial dose was continued. In cases with one or more dose changes, each dose was multiplied by its individual period and added to the other multiplied doses. Additionally, for bromocriptine patients we calculated the bromocriptine daily dose in milligrams and converted to a cabergoline-equivalent weekly dose, using for this conversion the maximal conventional doses of the medications as equivalent (bromocriptine 7.5 mg/day = cabergoline 2.0 mg/week) (18).

Statistical analysis was performed with SPSS version 25. Categorical variables were analyzed with cross tabulation using Chi-square test or Fisher's exact test where appropriate for small sample sizes, and odds ratios were calculated by 2 x 2 tables. Numeric variables between DA-treated and treatment naïve patients were tested with Mann-Whitney U if non-parametric or Student *t*-test if normally distributed. Both scoring systems were compared in a scatter plot using Pearson correlation and *r*/*r*^2^ were calculated for each group and the overall population.

## Results

Seventy-six DA-treated (functioning pituitary tumors) and 27 naïve (non-functioning or growth hormone-secreting pituitary tumors) patients were included in our analysis. In the overall sample, 73 patients had confirmed diagnosis of a prolactinoma, three had plurihormonal (prolactin and growth hormone) secreting tumors, 13 had non-functioning pituitary tumors (NFPA), and 14 had acromegaly (Table 1).

**Table 1:** Demographic characteristics and survey scores.

**Characteristic**	**DA (*n* = 76)**	**Control (*n* = 27)**	***P***
Sex female; *n* (%)	42 (55.3)	19 (70.4)	0.17°
Mean age; years ± (SD)	42.79 (15.56)	41.23 (15.09)	0.65^¥^
**Diagnosis;** ***n*** **(%)**			
• Prolactinoma	64 (84.2)	9 (33.3)	<0.001°
• NFPA	3 (3.9)	10 (37.0)	
• Acromegaly	6 (7.9)	8 (29.6)	
• Plurihormonal tumor	3 (3.9)	0 (0)	
Macroadenoma; *n* (%)	43 (56.6)	9 (33.3)	0.04°
Hypogonadism, male; *n* (%)	19 (55.9)	4 (50.0)	1.0*
Testosterone replacement; *n* (%)	3 (8.8)	2 (25.0)	0.23*
Prolactin; ng/ml (IQR)	11.3 (2.3–51.47)	18.0 (10.7–54.1)	0.04^¶^
Testosterone, male; ng/ml ± (SD)	318.1 (180.1)	306.2 (173.7)	0.86^¥^
Positive PHQ-9 Item 9; *n* (%)	6 (7.9)	2 (7.4)	0.648*
• “Several days” (1 point)	4 (5.3)	2 (7.4)	
• “Almost every day” (3 points)	2 (2.6)	0 (0)	
PHQ score; median (IQR)	4 (1–9)	5 (3–8)	0.36^¶^
Major Depression; *n* (%)	10 (13.2)	1 (3.7)	0.28°
Other Depression; *n* (%)	7 (9.2)	5 (18.5)	0.29°
**Severity by PHQ-9;** ***n*** **(%)**			
• Mild (5–9)	18 (23.7)	12 (44.4)	0.24°
• Moderate (10–14)	8 (10.5)	1 (3.7)	
• Moderately severe (15–19)	7 (9.2)	2 (7.4)	
• Severe (**≥**20)	3 (3.9)	0 (0)	
BIS-11 total score; mean ± (SD)	55 (11.8)	55.04 (10.42)	0.98^¥^
BIS-11 ≥ 60; *n* (%)	18 (26.5)	6 (23.1)	0.73°
BIS-11 **≥** 65; *n* (%)	14 (20.6)	5 (19.2)	0.88°
BIS-11 AI score; mean ± (SD)	14.63 (4.4)	14.38 (3.87)	0.80^¥^
BIS-11 MI score; mean ± (SD)	18.85 (4.19)	19.31 (3.94)	0.63^¥^
BIS-11 NPI score; mean ± (SD)	21.5 (5.48)	21.35 (4.58)	0.89^¥^

Data displayed as n (%), mean (± SD) or median (percentile 25th−75th).

IQR, Interquartile range.

° Chi square; *Fisher's Exact test;

¶ U Mann Whitney;

¥ *Student T*.

Cabergoline was used by most DA-treated patients (*n* = 70, 92.1%) with bromocriptine in six (*n* = 6, 7.9%). There was no significant difference in sex and age between DA-treated and naïve patients. Although 103 patients completed the PHQ-9 questionnaire, nine patients (8 DA-treated and 1 naïve) had missing responses to some BIS-11 questions and therefore, were excluded from analysis (*n* = 94; final analysis).

Minimal depression was found in 50.48% of the study patients and was 52.0% (*n* = 40) in DA-treated and 44.4% (*n* = 12) in DA treatment naïve patients. Mild depression was found in 29.1% of the study patients with a higher prevalence in DA treatment naïve patients 44.0% (*n* = 12) vs. 23.7% (*n* = 18) in DA-treated, which was not significant. Both moderate (8.7%) and moderately severe (8.7%) depression were more prevalent in DA-treated patients 10.5% (*n* = 8) and 9.2% (*n* = 7) respectively, vs. 3.7% (*n* = 1) and 7.4% (*n* = 2) of DA treatment naïve patients, which was not significant. Severe depression was only found in DA-treated patients 3.9% (*n* = 3). Two of these patients had thoughts of death or hurting themselves “nearly every day” as noted on item 9 of the PHQ-9.

Major depression was 10.7% overall and 13.2% (*n* = 10) in DA-treated patients, with a lower prevalence in naïve patients of 3.7% (*n* =1). Other depressive disorders were higher in the naïve patients 18.5% (*n* = 5) vs. DA-treated patients 9.2% (*n* = 7) and did not reach significance (*p* = 0.28). Higher PHQ-9 scores, major depression and other depressive disorders were not significantly associated with macroadenoma, male hypogonadism, testosterone levels or testosterone replacement.

A normal BIS-11 score (<60) was found in 76.7% (*n* = 79) of the study patients. However, 23.3% (26.5%; *n* = 18, DA-treated and 23.1%; *n* = 6, DA treatment naïve) had scores of ≥60 points, and 18.4% (20.6%; *n* = 14, DA-treated and 19.2%; *n* = 5, DA treatment naïve) had scores of ≥65 points. Although no significant difference was found, higher scores were found in DA-treated patients. Patients with BIS-11 scores of ≥60 points were younger and received lower cumulative DA doses compared to patients with BIS scores <60 (Table 2). There was no association between male sex and BIS-11 ≥ 60 and male sex did not increase odds for having increased point scores (OR = 0.66, CI 95% 0.25–1.76, *p* = 0.41). Higher BIS-11 scores were not associated with macroadenomas, male hypogonadism, testosterone levels or testosterone replacement. Treatment duration for those with BIS-11 scores of > 60 points almost reached significance (*p* = 0.054). The DA treatment naïve patient score of ≥60 points sample size (*n* = 6) could be the reason for this (Table 2).

**Table 2:** Association and correlation values for increased BIS-11 scores.

	**BIS-11 <60**	**BIS-11 ≥ 60**	***P***
Male	30 (78.9%)	8 (21.1%)	0.41° 0.28^+^
Female	40 (71.4%)	16 (28.6%)	
Hypogonadism, male	15 (50%)	4 (50%)	1.0^*^ 1.0^+^
Testosterone replacement, male	4 (13.3%)	1 (12.5%)	0.72^*^ 0.50^+^
Age (years)	43.7 (14.55)	35.9 (15.02)	0.02^¥^ 0.04^+^
Macroadenoma	19 (63.6%)	5 (62.5%)	0.63^*^ 0.46^+^
Prolactin (ng/ml)	11.0 (3–37.3)	22.7 (6.2–79.95)	0.09^¶^ 0.11^+^
PHQ-9 Score	3 (1–6)	10.5 (7.25–14.50)	<0.001^¶^ <0.001^+^
Dose Cab equivalent; mg/week (range)	1.0 (0.5–1.5)	1.0 (1.45–1.38)	0.35^¶^
Treatment duration; weeks (range)	94.98 (25.71–188.03)	27.71 (10.53–215.85)	0.05^¶^
Cumulative dose; mg (range)	120.46 (46.23–299.96)	18.27 (11.50–313.98)	0.02^¶^

Data displayed as n (%), mean (± SD) or median (percentile 25th−75th). IQR, Interquartile range.

° Chi square;

* Fisher's Exact test;

¶ U Mann Whitney;

¥ *Student T, ^+^p-value for DA-treated*.

**Figure 1:**
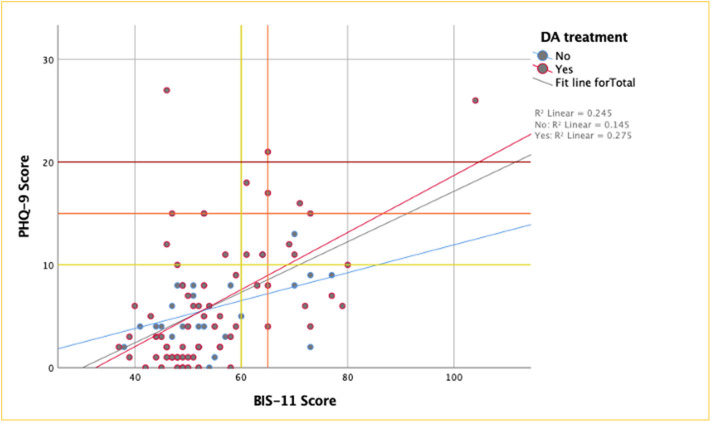
PHQ-9 by BIS-11 scores. Survey scores in patients treated with DA had a moderate correlation, reaching statistical significance, while correlation was mild and NS in the not treated group. Y-axis threshold lines: moderate (yellow), moderately severe (orange) and severe (red) depression by PHQ-9; X-axis reference lines: threshold for ICD patients with PRLs (yellow) & patients with gambling disorders (orange) by BIS-11.

We found 25.0% (*n* = 6/24) of patients with a BIS-11 score ≥ 60 also met the criteria for major depression, which means that patients with ICD have significantly increased odds of having a major depressive disorder (OR 5.5, CI 95% 1.4–21.6, *p* = 0.016). Impulse control disorder patients were found to have increased odds for having thoughts of death or self-harm (OR 5.8, CI 95% 1.2–26.8, *p* = 0.02). In addition, there was a positive correlation between higher BIS-11 and PHQ-9 scores, with a significantly higher correlation in DA-treated patients (*r* = 0.52, *p* <0.001), compared to DA treatment naïve patients (*r* = 0.32, *p* = 0.1) (Figure 1).

## Discussion

To our knowledge, this is the first study to evaluate DA-treated patients with pituitary disease using the PHQ-9 as a screening tool for depression and suicidal ideation performed routinely in a pituitary clinic setting.

Dopamine agonists are currently the cornerstone for prolactinoma and symptomatic hyperprolactinemia treatment (1). Dopamine agonists are also used for treatment of other non-pituitary diseases such as Parkinson's Disease and Restless Leg Syndrome (15, 19). In addition to the well-known side effects, such as nausea, dizziness, orthostatic hypotension, and headaches (7), which are usually preventable with slow up-titration in most clinical scenarios (15) some psychiatric side effects, such as ICD, have been noted (10, 19, 20). As such psychiatric side effects have the potential to impact patients' quality of life (QoL). Impulse control disorders have a wide range of clinical presentations, from punding to potentially risky behaviors, such as pathological gambling or hypersexuality (7). A new concern has emerged regarding the development of *de novo* ICD in patients treated with DA. To measure the magnitude of ICD in prolactinoma patients, several case control and observational studies have been undertaken. These studies have consistently reported a high incidence of ICD, but with unclear results concerning causation (12, 16, 21–23). These disorders do not seem dose-related; rather other mechanisms predispose certain patients (10).

Impulse control disorders are defined in the Diagnostic and Statistical Manual of Mental Disorders (DSM−5) as problems in self-control of emotions and behaviors, “manifested in behaviors that violate the rights of others and/or that bring the individual into significant conflict with societal norms or authority figures” (24). Impulse control disorders are much more common in males (10), though this can change when considering age and presence of another primary psychiatric disorder (25). Impulse control disorders and addictive disorders share a similar biological background, for example the decision to treat pathological gambling as an addictive disorder has crucial implications (26).

Impulsivity (27, 28) is a diagnostic criterion for many psychiatric disorders, namely ICD. Furthermore, impulsivity has been associated with a predisposition to engage in risky behaviors and is particularly relevant for depression and suicidal behavior. It has been reported that when impulsivity and suicidal ideation are combined, the risk for acting on those suicidal ideas is increased (11).

Pathophysiology of ICD in DA treated patients remains unclear. It has been hypothesized that a faulty top-down control involving dopaminergic, serotoninergic, and GABA(γ-aminobutyric acid) neurotransmission in the orbitofrontal and anterior cingulate cortices in addition to bottom-up control by limbic structures is contributory (25, 29). It has been proposed that DA may predispose individuals to impulsivity through an abatement of negative reinforcement feedback learning, and through abnormal dopaminergic signaling in the mesolimbic and mesocortical pathways (10).

Currently, instruments/tools available to measure ICD are limited and clinical utility is not well established. This holds true in particular in the context of DA therapy. The BIS-11 is the most widely used validated scale for assessment of impulsivity. Specifically, for an ICD diagnosis, the only available tool is the recently developed and yet to be validated for routine use by clinicians; Minnesota Impulse Disorders Interview (MIDI) (30). Other tools such as the clinical version of the Structured Clinical Interview for DSM Disorders (SCID-5-CV) (31), Mini International Neuropsychiatric Interview (MINI) include questionnaires for the detection of Antisocial Personality Disorder, and addictive disorders (32). The Questionnaire for Impulsive-Compulsive Disorders in Parkinson's Disease (QUIP) (33) is of limited use in a clinical setting. Other scales, such as The Hypersexual Behavior Inventory (HBI), the Hypersexual Behavior Consequences Scale (HBCS), and the Social Desirability Response Set Scale (SDRSS) only assess the presence and severity of hypersexuality (34–36).

Depression is one of the most common psychiatric disorders in the general population, globally (37). People who are chronically ill are especially prone to depression because of the intrinsic nature of the medical disease, the use of medications with behavioral and cognitive side effects, and the psychosocial burden that accompanies the process of falling ill and living with the disease (38). While there are a number of clinical scales available for the screening of depression in medically ill patients, such as the Beck Depression inventory (BDI), Hospital Anxiety and Depression Scale, and the PHQ-9, a complete mental health assessment is still not routine in most clinical scenarios. This can have deleterious effects on patients' general well-being, as untreated depression in medical settings is associated with worsened health outcomes in the chronically ill (39). On the other hand, it has been demonstrated that the timely diagnosis and treatment of depression in the medically ill population is time and cost effective (40) and associated with better health outcomes (41).

Here in patients preselected for absence of known depression (patients with history of depression did not get a survey) we did not find significant differences in ICD prevalence amongst patients treated with and naïve to DA. However, our aim was to screen patients for ICD using a brief test; the BISS-11, which is suitable for use during clinic visits (12, 16). Prevalence of ICD in the literature is controversial. Multiple tools have been used to identify ICD, such as the MIDI, South Oaks Gambling Screen, modified hypersexuality and punding questionnaires, QUIP and SDRSS, among others. We found an ICD prevalence of 23.3% in the DA-treated patients, which is higher (10%, *n* = 2) (21), similar (24.6 and 17%, respectively), (16, 22, 23) and lower than other studies (61%) (16, 22, 23). The only prospective 1-year follow up study (12) found an incidence of 8% of new-onset ICD as measured by MIDI and BIS-11 among prolactinoma DA-treated patients.

In contrast with previous studies that reported male predominance in patients developing ICD, we show that prolactin levels and male sex were not associated with a diagnosis of ICD (based on both BIS score >60 and >65) (22). Consistent with Bancos et al., we found lack of association of macroadenomas and increased impulsivity scores (22). Additionally, no association was found between male hypogonadism, testosterone levels or testosterone replacement in men with increased impulsivity scores. Higher testosterone levels at the last visit in male patients developing hypersexuality were reported by another study (16), which is not specifically sought by BIS-11, but testosterone levels or replacement were not associated with increased impulsivity in our study. An increased ICD risk, especially hypersexuality, in men with normal testosterone has been also noted previously (23), which we did not find. One possible explanation is the cross-sectional design of our study may have missed an “acute increase” in testosterone levels, which may have transiently increased impulsivity. Furthermore, we found for the first time that younger patients had significantly higher ICD scores (BIS score > 60). Patients with increased ICD scores had a mean age of 35 years, which is around 8 years younger than patients with normal scores. Additionally, those patients with a lower cumulative dose were more likely to be diagnosed with an ICD according to BIS-11, (possibly explained by an increased sensitivity to DA agents' psychiatric side effects in patients who are DA naïve and start treatment) (10). The high percentage of co-existing ICD and depression cases among DA-treated patients was an unexpected finding and the combination of depressive symptoms and ICD is alarming because it could prompt an increased risk of suicidality (11). Therefore, careful consideration of specific intervention for patients who have suicidal or self-harming thoughts in combination with ICD is warranted. We suggest psychiatric counseling and multidisciplinary follow- up in patients with abnormal scores before starting DAs or increasing doses.

A study limitation is the diagnostic heterogeneity in DA-treated and DA treatment naïve patients and the smaller number of DA treatment naïve patients. This falls short of clarifying the potential role of primary disease as a risk factor in the development of psychiatric symptoms when treated with DA. Both PHQ-9 and BIS-11 are brief tools, which cannot identify other diagnoses beside depression and ICD, and BIS-11 has a variable cutoff value, depending on specific group characteristics. We also preselected patients without a known history of depression, which can theoretically lower the calculated prevalence.

Study strengths include standardized use in a large consecutive sample of patients in a real-life clinical setting for management of pituitary disease, and the selection of effective and easy to apply tools to screen depression and ICD.

Overall, we consider that PHQ-9 and BIS-11 assessments are acceptable screening tools in the absence of specific questionnaires for the diagnosis of ICD and depression, specifically, Item 9 of the PHQ-9, which is related to thoughts of self-harm and suicidal ideation. The potential cost of missing such a statement during a medical assessment warrants the need to implement routine screening for depressive symptoms and suicidal ideation/behavior.

## Conclusion

We show for the first time that a combination of PHQ-9 and BIS-11 questionnaires is practical and acceptable for routine screening of depression and ICD in patients with pituitary disease during routine outpatient pituitary clinic visits. Dopamine agonist therapy has a high, previously underestimated risk of ICD and coexistence of depression and ICD is likely to occur more frequently in patients with pituitary disease when treated with DA. We suggest close follow-up after initiation of DA treatment in younger patients, regardless of dose. Early detection of depression and ICD and timely discontinuation of DA medications may prevent harmful consequences. As the psychiatric effects of DA use are increasingly recognized and understood, further research is needed. Likewise, is the development of reliable tools for depression and ICD diagnosis and classification.

## Data Availability Statement

The data supporting the conclusions of this article will be made available by the authors, under advisement of the Institutional Review Board.

## Ethics Statement

The studies involving human participants were reviewed and approved by Oregon Health & Science University Institutional Review Board. Written informed consent for participation was not required for this study in accordance with the national legislation and the institutional requirements.

## Author Contributions

MF: conception and design, acquisition of data, analysis and interpretation of data, drafting the article, critically revising the article, reviewed submitted version of manuscript, study supervision, IRB submission and approval. JH-A: conception and design, acquisition of data, analysis and interpretation of data, drafting the article, critically revising the article, reviewed submitted version of manuscript, statistical analysis, administrative/technical/material support, study supervision. NJ: acquisition of data, analysis and interpretation of data, reviewed submitted version of manuscript. CG-T: analysis and interpretation of data, drafting the article, critically revising the article, reviewed submitted version of manuscript, statistical analysis. EVV: conception and design, acquisition of data, analysis and interpretation of data, drafting the article, critically revising the article, reviewed submitted version of manuscript, statistical analysis. CGY: acquisition of data, analysis and interpretation of data, drafting the article, critically revising the article, reviewed submitted version of manuscript, statistical analysis. SM: analysis and interpretation of data, drafting the article, critically revising the article, reviewed submitted version of manuscript, administrative/technical/material support, IRB submission and approval. All authors contributed to the article and approved the submitted version.

## Conflict of Interest

The authors declare that the research was conducted in the absence of any commercial or financial relationships that could be construed as a potential conflict of interest.

## References

[1] Melmed S Casanueva FF Hoffman AR Kleinberg DL Montori VM Schlechte JA. Diagnosis and treatment of hyperprolactinemia: an endocrine society clinical practice guideline. J Clin Endocrinol Metab. (2011) 96:273–88. 10.1210/jc.2010-1692 21296991

[2] Melmed S Pituitary-tumor endocrinopathies. New Engl J Med. (2020) 382:937–50. 10.1056/NEJMra1810772 32130815

[3] Melmed S. Mechanisms for pituitary tumorigenesis: the plastic pituitary. J Clin Investig. (2003) 112:1603–18. 10.1172/JCI20401 14660734 PMC281651

[4] Liu X Tang C Wen G Zhong C Yang J Zhu J. The mechanism and pathways of dopamine and dopamine agonists in prolactinomas. Front Endocrinol. (2019) 9:768. 10.3389/fendo.2018.00768 30740089 PMC6357924

[5] Liu W Zahr RS Mccartney S Cetas JS Dogan A Fleseriu M. Clinical outcomes in male patients with lactotroph adenomas who required pituitary surgery: a retrospective single center study. Pituitary. (2018) 21:454–62. 10.1007/s11102-018-0898-y 29936681

[6] Klibanski A. Prolactinomas. New Engl J Med. (2010) 362:1219–26. 10.1056/NEJMcp0912025 20357284

[7] Ioachimescu AG Fleseriu M Hoffman AR Vaughan TB Iii Katznelson L. Psychological effects of dopamine agonist treatment in patients with hyperprolactinemia and prolactin-secreting adenomas. Eur J Endocrinol. (2019) 180:31–40. 10.1530/EJE-18-0682 30400048

[8] Colao A. Improvement of cardiac parameters in patients with acromegaly treated with medical therapies. Pituitary. (2011) 15:50–8. 10.1007/s11102-011-0318-z 21713528

[9] Hinojosa-Amaya JM Cuevas-Ramos D Fleseriu M. Medical management of cushing's syndrome: current and emerging treatments. Drugs. (2019) 79:935–56. 10.1007/s40265-019-01128-7 31098899

[10] Grall-Bronnec M Victorri-Vigneau C Donnio Y Leboucher J Rousselet M Thiabaud E. Dopamine agonists and impulse control disorders: a complex association. Drug Safety. (2018) 41:19–75. 10.1007/s40264-017-0590-6 28861870 PMC5762774

[11] Mann JJ Waternaux C Haas GL Malone KM. Toward a clinical model of suicidal behavior in psychiatric patients. Am J Psychiatry. (1999) 156:181–9. 9989552 10.1176/ajp.156.2.181

[12] Celik E Ozkaya HM Poyraz BC Saglam T Kadioglu P. Impulse control disorders in patients with prolactinoma receiving dopamine agonist therapy: a prospective study with 1 year follow-up. Endocrine. (2018) 62:692–700. 10.1007/s12020-018-1744-8 30206771

[13] Kroenke K Spitzer RL Williams JB. The PHQ-9: validity of a brief depression severity measure. J Gen Int Med. (2001) 16:606–13. 10.1046/j.1525-1497.2001.016009606.x 11556941 PMC1495268

[14] Louzon SA Bossarte R Mccarthy JF Katz IR. Does suicidal ideation as measured by the PHQ-9 predict suicide among VA patients? Psychiatr Serv. (2016) 67:517–22. 10.1176/appi.ps.201500149 26766757

[15] Barake M Klibanski A Tritos NA. Management Of Endocrine Disease: Impulse control disorders in patients with hyperpolactinemia treated with dopamine agonists: how much should we worry? Eur J Endocrinol. (2018) 179:R287–96. 10.1530/EJE-18-0667 30324793

[16] Dogansen SC Cikrikcili U Oruk G Kutbay NO Tanrikulu S Hekimsoy Z. Dopamine agonist-induced impulse control disorders in patients with prolactinoma: a cross-sectional multicenter study. J Clin Endocrinol Metab. (2019) 104:2527–34. 10.1210/jc.2018-02202 30848825

[17] Marazziti D Baroni S Picchetti M Catena Dell'osso M Impulsivity in pathological gambling. Eur Psychiatry. (2011) 26:1734 10.1016/S0924-9338(11)73438-1

[18] Molitch ME. Management of medically refractory prolactinoma. J Neurooncol. (2014) 117:421–8. 10.1007/s11060-013-1270-8 24146188

[19] Cornelius JR Tippmann-Peikert M Slocumb NL Frerichs CF Silber MH. Impulse control disorders with the use of dopaminergic agents in restless legs syndrome: a case-control study. Sleep. (2010) 33:81–7. 20120624 PMC2802252

[20] Weintraub D Siderowf AD Potenza MN Goveas J Morales KH Duda JE. Association of dopamine agonist use with impulse control disorders in Parkinson disease. Arch Neurol. (2006) 63:969–73. 10.1001/archneur.63.7.969 16831966 PMC1761054

[21] Martinkova J Trejbalova L Sasikova M Benetin J Valkovic P. Impulse control disorders associated with dopaminergic medication in patients with pituitary adenomas. Clin Neuropharmacol. (2011) 34:179–81. 10.1097/WNF.0b013e3182281b2f 21738024

[22] Bancos I Nannenga MR Bostwick JM Silber MH Erickson D Nippoldt TB. Impulse control disorders in patients with dopamine agonist-treated prolactinomas and nonfunctioning pituitary adenomas: a case-control study. Clin Endocrinol. (2014) 80:863–8. 10.1111/cen.12375 24274365 PMC4136510

[23] De Sousa SMC Baranoff J Rushworth RL Butler J Sorbello J Vorster J. Impulse control disorders in dopamine agonist-treated hyperprolactinemia: prevalence and risk factors. J Clin Endocrinol Metab. (2019) 105:e108–18. 10.1210/clinem/dgz076 31580439

[24] American Psychiatric Association Diagnostic and statistical manual of mental disorders. Am Psychiatr Assoc. (2013). 10.1176/appi.books.9780890425596

[25] Kulacaoglu F Kose S Singing under the impulsiveness: impulsivity in psychiatric disorders. Psychiatr Clin Psychopharmacol. (2017) 28:205–10. 10.1080/24750573.2017.1410329

[26] Clark L (2015). From Impulsivity to Addiction: Gambling Disorder and Beyond. Available online at: https://www.psychiatrictimes.com/view/impulsivity-addiction-gambling-disorder-and-beyond (accessed May 1, 2020).

[27] Bakhshani N-M. Impulsivity: a predisposition toward risky behaviors. Int J High Risk Behav Addict. (2014) 3:e20428. 10.5812/ijhrba.20428 25032165 PMC4080475

[28] Hilbert A Binge-eating disorder. Psychiatr Clin N Am. (2019) 42:33–43. 10.1016/j.psc.2018.10.011 30704638

[29] Leshem R Glicksohn J The construct of impulsivity revisited. Person Indiv Differ. (2007) 43:681–91. 10.1016/j.paid.2007.01.015

[30] Chamberlain SR Grant JE. Minnesota impulse disorders interview (MIDI): validation of a structured diagnostic clinical interview for impulse control disorders in an enriched community sample. Psychiatry Res. (2018) 265:279–83. 10.1016/j.psychres.2018.05.006 29772488 PMC5985960

[31] First MB Structured clinical interview for theDSM(SCID). In: Cautin RL and Lilienfeld SO, editors. The Encyclopedia of Clinical Psychology. John Wiley & Sons, Inc. (2015). p. 1–6. 10.1002/9781118625392.wbecp351

[32] Sheehan DV Lecrubier Y Sheehan KH Amorim P Janavs J Weiller E. The Mini-International Neuropsychiatric Interview (M.I.N.I.): the development and validation of a structured diagnostic psychiatric interview for DSM-IV and ICD-10. J Clin Psychiatry. (1998) 59 (Suppl. 20):22-33;quiz 34-57. 9881538

[33] Weintraub D Hoops S Shea JA Lyons KE Pahwa R Driver-Dunckley ED. Validation of the questionnaire for impulsive-compulsive disorders in Parkinson's disease. Mov Disord. (2009) 24:1461–7. 10.1002/mds.22571 19452562 PMC2848971

[34] Hays RD Hayashi T Stewart AL A five-item measure of socially desirable response set. Educ Psychol Meas. (1989) 49:629–36. 10.1177/001316448904900315

[35] Reid RC Garos S Fong T. Psychometric development of the hypersexual behavior consequences scale. J Behav Addict. (2012) 1:115–22. 10.1556/JBA.1.2012.001 26165461

[36] Bothe B Kovács M Tóth-Király I Reid RC Griffiths MD Orosz G. The psychometric properties of the hypersexual behavior inventory using a large-scale nonclinical sample. J Sex Res. (2018) 56:180–90. 10.1080/00224499.2018.1494262 30028633

[37] World Health Organization Depression and Other Common Mental Disorders Global Health Estimates. (2017). Available online at: https://www.who.int/mental_health/management/depression/prevalence_global_health_estimates/en/ (accessed May 1, 2020).

[38] Levenson JL *The* American Psychiatric Association Publishing *Textbook of Psychosomatic Medicine and Consultation-Liaison Psychiatry*. Washington, DC: American Psychiatric Association Publishing (2018).

[39] Moussavi S Chatterji S Verdes E Tandon A Patel V Ustun B Depression, chronic diseases, and decrements in health: results from the World Health Surveys. Lancet. (2007) 370:851–8. 10.1016/S0140-6736(07)61415-9 17826170

[40] Blount A Schoenbaum M Kathol R Rollman BL Thomas M O'donohue W The economics of behavioral health services in medical settings: A summary of the evidence. Prof Psychol Res Pract. (2007) 38:290–7. 10.1037/0735-7028.38.3.290

[41] Schoenbaum M Unützer J Mccaffrey D Duan N Sherbourne C Wells KB. The effects of primary care depression treatment on patients' clinical status and employment. Health Serv Res. (2002) 37:1145–58. 10.1111/1475-6773.01086 12479490 PMC1464020

