# Supplementary material for: Depression and Impulsivity Self-Assessment Tools to Identify Dopamine Agonist Side Effects in Patients With Pituitary Adenomas
Source: Front Endocrinol (Lausanne). 2020 Oct 27;11:579606. doi: 10.3389/fendo.2020.579606 (PMC7652723; doi:10.3389/fendo.2020.579606)
Supplement: Supplementary file 1 [file Table_1.DOCX]

**Supplemental Table 1.** Patient Health Questionnaire 9 (PHQ-9) Questionnaire*

| Over the last 2 weeks how often have you been bothered by any of the following problems? | Not at all | Several  days | More than  half the days | Nearly  everyday |
| --- | --- | --- | --- | --- |
| 1. Little interest or pleasure in doing things | 0 | 1 | 2 (S) | 3 (S) |
| 2. Feeling down, depressed, or hopeless | 0 | 1 | 2 (S) | 3 (S) |
| 3. Trouble falling or staying asleep, or sleeping too much | 0 | 1 | 2 (S) | 3 (S) |
| 4. Feeling tired or having little energy | 0 | 1 | 2 (S) | 3 (S) |
| 5. Poor appetite or overeating | 0 | 1 | 2 (S) | 3 (S) |
| 6. Feeling bad about yourself − or that you are a failure or have let yourself or your family down | 0 | 1 | 2 (S) | 3 (S) |
| 7. Trouble concentrating on things, such as reading the newspaper or watching television | 0 | 1 | 2 (S) | 3 (S) |
| 8. Moving or speaking so slowly that other people could have noticed?  Or the opposite − being so fidgety or restless that you have been moving around a lot more than usual | 0 | 1 | 2 (S) | 3 (S) |
| 9. Thoughts that you would be better off dead or of hurting yourself in some way | 0 | 1 (S) | 2 (S) | 3 (S) |
| Add Items above | | | | |
| If you checked off any problems, how difficult have these problems made it for you to do your work, take care of things at home, or get along with other people? | Not difficult  at all | Somewhat  difficult | Very  difficult | Extremely  difficult |

*The copyright for the PHQ-9 was formerly held with Pfizer, who provided the educational grant for Drs Spitzer, Williams and Kroenke who originally designed it. This is no longer the case and no permission is required to reproduce, translate, display or distribute the PHQ-9.
