# Supplementary material for: Depression and Impulsivity Self-Assessment Tools to Identify Dopamine Agonist Side Effects in Patients With Pituitary Adenomas
Source: Front Endocrinol (Lausanne). 2020 Oct 27;11:579606. doi: 10.3389/fendo.2020.579606 (PMC7652723; doi:10.3389/fendo.2020.579606)
Supplement: Supplementary file 2 [file Table_2.DOCX]

**Supplemental Table 2.** Patient Health Questionnaire 9 (PHQ-9) interpretation criteria

| **Criteria for depressive disorders** | |
| --- | --- |
| Major Depressive Disorder | If there are at least 5 checks in the shaded (S) sections.  One corresponds to Question #1 or #2 |
| Other Depressive Disorders | If there are 2 – 4 checks in the shaded (S) sections.  One corresponds to Question #1 or #2 |
| **Total Score Interpretation** | |
| 1 – 4 | Minimal depression |
| 5 – 9 | Mild depression |
| 10 – 14 | Moderate depression |
| 15 – 19 | Moderately severe depression |
| 20 – 27 | Severe depression |
