# Supplementary material for: Depression and Impulsivity Self-Assessment Tools to Identify Dopamine Agonist Side Effects in Patients With Pituitary Adenomas
Source: Front Endocrinol (Lausanne). 2020 Oct 27;11:579606. doi: 10.3389/fendo.2020.579606 (PMC7652723; doi:10.3389/fendo.2020.579606)
Supplement: Supplementary file 3 [file Table_3.DOCX]

**Supplemental Table 3.** Barratt Impulsivity Scale (BIS-11)*

| DIRECTIONS: People differ in the ways they act and think in different situations.  This is a test to measure some of the ways in which you act and think.  Read each statement and put an X on the appropriate circle on the right side of this page. | | | | |
| --- | --- | --- | --- | --- |
| Do not spend too much time on any statement.  Answer quickly and honestly. | 1  Rarely/ Never | 2  Occasionally | 3  Often | 4  Almost Always/Always |
| 1.- I plan tasks carefully. | ○ | ○ | ○ | ○ |
| 2.- I do things without thinking. | ○ | ○ | ○ | ○ |
| 3.- I make-up my mind quickly. | ○ | ○ | ○ | ○ |
| 4.- I am happy-go-lucky. | ○ | ○ | ○ | ○ |
| 5.- I don’t “pay attention.” | ○ | ○ | ○ | ○ |
| 6.- I have “racing” thoughts. | ○ | ○ | ○ | ○ |
| 7.- I plan trips well ahead of time. | ○ | ○ | ○ | ○ |
| 8.- I am self-controlled. | ○ | ○ | ○ | ○ |
| 9.- I concentrate easily. | ○ | ○ | ○ | ○ |
| 10.- I save regularly. | ○ | ○ | ○ | ○ |
| 11.- I “squirm” at plays or lectures. | ○ | ○ | ○ | ○ |
| 12.- I am a careful thinker. | ○ | ○ | ○ | ○ |
| 13.- I plan for job security. | ○ | ○ | ○ | ○ |
| 14.- I say things without thinking. | ○ | ○ | ○ | ○ |
| 15.- I like to think about complex problems. | ○ | ○ | ○ | ○ |
| 16.- I change jobs. | ○ | ○ | ○ | ○ |
| 17.- I act “on impulse.” | ○ | ○ | ○ | ○ |
| 18.- I get easily bored when solving thought problems. | ○ | ○ | ○ | ○ |
| 19.- I act on the spur of the moment. | ○ | ○ | ○ | ○ |
| 20.- I am a steady thinker. | ○ | ○ | ○ | ○ |
| 21.- I change residences. | ○ | ○ | ○ | ○ |
| 22.- I buy things on impulse. | ○ | ○ | ○ | ○ |
| 23.- I can only think about one thing at a time. | ○ | ○ | ○ | ○ |
| 24.- I change hobbies. | ○ | ○ | ○ | ○ |
| 25.- I spend or charge more than I earn. | ○ | ○ | ○ | ○ |
| 26.- I often have extraneous thoughts when thinking. | ○ | ○ | ○ | ○ |
| 27.- I am more interested in the present than the future. | ○ | ○ | ○ | ○ |
| 28.- I am restless at the theater or lectures. | ○ | ○ | ○ | ○ |
| 29.- I like puzzles. | ○ | ○ | ○ | ○ |
| 30.- I am future oriented. | ○ | ○ | ○ | ○ |

*Barrat ES. Anxiety and impulsiveness related to psychomotor efficiency. Perceptual and Motor Skills; 1959; 9:191-198

*Patton JH, Stanford MS, Barratt ES. Factor structure of the Barratt impulsiveness scale. J Clin Psychol. 1995 Nov;51(6):768-74

*Stanford MS, Mathias CW, Dougherty DM, Lake SL, Anderson NE, and Patton JH. Fifty years of the Barratt Impulsiveness Scale: An update and review.

Personality and Individual Differences,2009; 47:385-395
