# Supplementary material for: Depression and Impulsivity Self-Assessment Tools to Identify Dopamine Agonist Side Effects in Patients With Pituitary Adenomas
Source: Front Endocrinol (Lausanne). 2020 Oct 27;11:579606. doi: 10.3389/fendo.2020.579606 (PMC7652723; doi:10.3389/fendo.2020.579606)
Supplement: Supplementary file 4 [file Table_4.DOCX]

**Supplemental Table 4.** Barratt Impulsivity Scale (BIS-11) factor structure and scoring.

| **Second order factors** | **First order factors** | **Number of items** | **Items contributing to each subscale** |
| --- | --- | --- | --- |
| Attentional | Attention | 5 | 5, 9*, 11, 20*, 28 |
|  | Cognitive Instability | 3 | 6, 24, 26 |
| Motor | Motor | 7 | 2, 3, 4, 17, 19, 22, 25 |
|  | Perseverance | 4 | 16, 21, 23, 30* |
| Non-planning | Self-Control | 6 | 1*, 7*, 8*, 12*, 13*, 14 |
|  | Cognitive Complexity | 5 | 10*, 15*, 18, 27, 29* |
| *reverse scored items | | | |
